# Feasibility study for the non-invasive blood pressure estimation based on ppg morphology: normotensive subject study

**DOI:** 10.1186/s12938-016-0302-y

**Published:** 2017-01-10

**Authors:** Hangsik Shin, Se Dong Min

**Affiliations:** 1 Healthcare Solution Laboratory, Department of Biomedical Engineering, Chonnam National University, 502, 3rd Eng. Bldg., 50 Daehak-ro, Yeosu, South Korea; 2 Department of Medical IT Engineering, College of Medical Science, Soonchunhyang University, 22, Soonchunhyang-ro, Eumnae-ri, Sinchang-myeon, Asan-si, Chungcheongnam-do Republic of Korea

**Keywords:** Photoplethysmography (PPG), Derivative photoplethysmography, Systolic blood pressure, Pulse pressure, Vessel wall movement

## Abstract

**Background:**

Blood pressure is a critical bio-signal and its importance has been increased with the aged society and the growth of cardiovascular disease population. However, most of hypertensive patients have been suffered the inconvenience in monitoring blood pressure in daily life because the measurement of the blood pressure depends on the cuff-based technique. Nowadays there are many trials to measure blood pressure without cuff, especially, photoplethysmography (PPG) based research is carried out in various ways.

**Methods:**

Our research is designed to hypothesis the relationship between vessel wall movement and pressure-flow relationship of PPG and to validate its appropriateness by experimental methods. PPG waveform is simplified by approximate model, and then it is analyzed as the velocity and the acceleration of blood flow using the derivatives of PPG. Finally, we develop pressure index (PI) as an estimation factor of blood pressure by combining of statistically significant segments of photoplethysmographic waveform.

**Results:**

Twenty-five subjects were participated in the experiment. As a result of simulation, correlation coefficients between developed PI and blood pressure were represented with *R* = 0.818, *R* = 0.827 and *R* = 0.615 in systolic blood pressure, pulse pressure and mean arterial pressure, respectively, and both of result showed the meaningful statistically significance (*P* < 0.05).

**Conclusions:**

Current study can estimate only the relative variation of blood pressure but could not find the absolute pressure value. Moreover, proposed index has the limitation of diastolic pressure tracing. However, the result shows that the proposed PI is statistically significantly correlated with blood pressures, and it suggests that the proposed PI as a promising additional parameter for the cuff less blood pressure monitoring.

## Background

Arterial blood pressure (ABP) is a very important clinical parameter, and numerous attempts have been made for continuous non-invasive measurements of ABP. The term of blood pressure (BP) usually refers to brachial arterial pressure because major vessels are located on the upper left or right arm to take blood away from the heart, moreover brachial pressure measurement has an advantage of non-invasive measurement. BP waveform analysis and synthesis have been investigated from BP and flow analysis, and it is the general consensus that BP waveform is consisted with the combination of the incident wave transmitted directly from the left ventricle to the finger and the reflected wave from the sites of impedance mismatch mainly in lower body [1, 2].

Several observations demonstrate that the amplitude and timing of wave reflections are directly related to the elastic properties of the arterial tree, stiffness index and time delay between the incident and reflected wave peaks are an example to estimate arterial stiffness [3]. The contour of the ascending aortic pressure wave has been classified by analyzing the reflected wave amplitude and temporal characteristics [4–6]. These classifications, however, are in close agreement with the age-related four classes of photoplethysmography (PPG) contour [7]. Moreover, it was demonstrated the age-related trend towards PPG contour triangulation [8], and showed the similar shape changes compared with the pressure wave. These results imply that PPG contour is dominantly controlled by pressure waveform, and contains cardiovascular information which includes vessel stiffness and BP.

Morphological analysis of PPG has been applied in vascular assessment such as vascular disease [9–11], aging [7, 8, 12, 13], and arterial compliance [14]. Though PPG morphologies have been provided abundant information for cardiovascular analysis, it is difficult to find literatures of BP estimation method performed by PPG morphology characteristic analysis. In most of previous researches, PPG has been used for BP measurement, not as a separated method of its waveform characteristics, but as a tool for the detection of blood volume change related to other devices in specific conditions.

PPG-based non-invasive BP monitoring may be a promising, however, it has not allowed for the clinical application at this time because there still are lacking points for estimation BP from PPG [15]. Most of PPG-based BP estimations were based on surrogate pulse measures of BP, which includes tracking beat-to-beat changes in pressure using the pulse transit time (PTT) [16–20] or pulse arrival time (PAT) [21–23] and PTT or PAT was usually calculated between the ECG-R wave and the foot of the PPG waveform for analysis. In recent research which based on deep belief network restricted Boltzmann machine (DBN-RBM), PPG-based BP estimation shows inadequate performance in BP estimation from intrinsic variability and wide limits of agreement [15]. Another research, which estimates BP by combining PTT and various PPG morphology characteristic such as PTT, time ratio of systole to diastole, area ratio of systole to diastole, time span of PPG cycle and diastolic duration, combined analysis, confirmed that the morphological characteristic could improve the accuracy of BP estimation. However, it also contains clinically significant errors [24].

Modified volume-oscillometric technique [25, 26] and hydrostatic method [27] is another BP estimation technique based on PPG sensor and two micro-electro mechanical systems (MEMS) accelerometers. Finapres™ (FINger Arterial PRESsure) and Portapres™ technology, which are regarded as a representative PPG-based BP measurement method [28, 29], provides continuous non-invasive BP recording from finger and is widely used, however it substantially measures BP not by PPG waveform but by volume-clamp method. Finger pressure is actually measured by finger cuff, and PPG is used as an auxiliary device to check whether blood volume is changed or not.

Because PPG waveform means the amount of blood in measuring spot and amount of blood is closely related to blood flow, PPG waveform should be influenced by pressure waveform which generates flow. Moreover, many of PPG applications are related to the angiological analysis of blood vessel [30]. From these characteristics of PPG, in this literature, we postulated that PPG could contain BP index and it may be related to blood vessel movements. In investigating blood vessel movement and PPG waveform, first derivative and second derivative PPG was applied to consider of flow-pressure relationship. It was proposed the first derivative-based flow waveform derivation method [31] and demonstrated derived flow has a very similar shape compared with Doppler flow waveforms [32]. Second derivation PPG, usually referred as the second derivative of the photoplethysmogram waveform (SDPTG) [13], means the acceleration of blood volume changes, and it means instantaneous power of blood circulation.

The proposed method aims to enhance BP estimation from PPG by using analyzing of PPG morphology and inspection of BP-related features. Our study was designed to (1) analyze first and second derivative PPG waveform and find the meaning of the hemodynamic changes, (2) set up the proper model to extract pressure-related parameters, and (3) assess whether derived indexes can help to identify measured BP with experimental data.

## Methods

Generally, PPG was measured the reflected or transmitted signal at a minute spot, in other words pressure gradient could be approximated with the derivative of pressure in PPG measurement. Moreover, it has been regarded as the second derivative as acceleration PPG and it implies the rate of change of pressure components in PPG [33]. To validate of derivative characteristics and BP relationship, modeling and evaluation was performed by approximated modeling, derivative analysis and experimental assessment in order.

### Vessel wall movement and approximated model

**Fig. 1:**
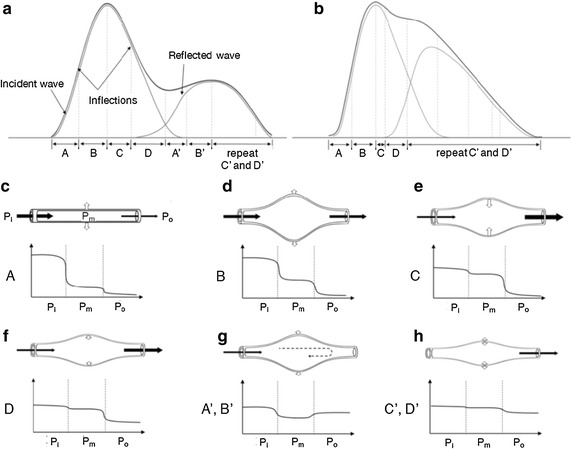
An example of an approximated model of conceptual PPG waveform and vessel wall movements. **a** An example of approximated model of **a** normotensive waveform (SBP 115 mmHg, subject no. 9, **b** pre-hypertensive waveform (SBP 137 mmHg, subject no. 11). **A, B, C, D, A**′**, B**′**, C**′ and **D**′ are classified by vessel wall movement, and Pi, Pm and Po mean input, measurement spot and output pressure respectively. **c** Early-systole (period **A**), **d** late-systole (period **B**), (**e**) early-diastole (period **C**), **f** late-diastole and start of reflected wave (period **D**), **g** early-reflection and P_o_ > P_i_ in normotensive case (period **A**′ and **B**′), **h** late-reflection (period **C**′ and **D**′), multiple reflected waves are arrived in order, period **C**′ and **D**′ are represented alternately

The reflected wave is represented as not a single pulse wave, but a multiple waves which are less sharply peaked and more spread out in time. It was already showing the pressure wave propagation with completely and incompletely occluded tube, and multiple reflected waves were found in both cases [34]. PPG waveform is also influenced by multiple reflected waves, however, no reflected wave could have an influence to incident wave but first reflected wave. Except on first reflected wave, reflected waves used to be found in the latter decreases and these waves disturb the incident wave analysis. Therefore PPG waveform is reconstructed with incident wave and first reflected wave in approximated model. The approximated model could not represent PPG waveform exactly, but it is helpful in the macroscopic analysis of pressure changes. Figure 1 shows the approximated model for conceptual of PPG waveform. Figure 1a represents normotensive PPG waveform and b shows the waveform in hypertension.

According to blood flow, blood vessel wall moves. It was observed by Doppler method that pulsatile flow, which is generated by heart cycle, directly affect vasoconstriction and vasodilatation [35]. The blood vessel is an elastic tube. Thus, inner volumes and vessel diameter could be varied by pressure gradients. Figure 1c–h represents vessel wall movement and pressure difference of the input (P_i_), measurement spot (P_m_) and output (P_o_). The systolic pressure is propagated to vessel in the early-systole period (Fig. 1c), and blood volume increases rapidly because pressure difference is large between P_i_ and P_m_. Blood volume is also increased in the late-systole period (Fig. 1d), however the blood volume change rate is decreased by the diminishing of the pressure gradients. Figure 1e, f shows early-diastole and late-diastole vessel wall movement respectively. In early-diastole, the pressure gradient is increased between P_m_ and P_o_, and rapid outflow is occurred. Outflow is diminished by decreasing of the pressure gradient between P_m_ and P_o_. Figure 1g, h shows that effect of the reflected wave. P_o_ is increased by reflected wave, and it causes the decrease of output pressure gradients. This change suppresses output flow, thus increasing of blood volume at measurement spot. Pressure gradient inversion causes late-upward peak as the case may be (Fig. 1g). Multiple reflect waves are successively arrived, and P_o_ and blood volume are fluctuated by each wave in late-reflection period (Fig. 1h). In the approximated model, late-reflection period is ignored.

### Derivative analysis

**Fig. 2:**
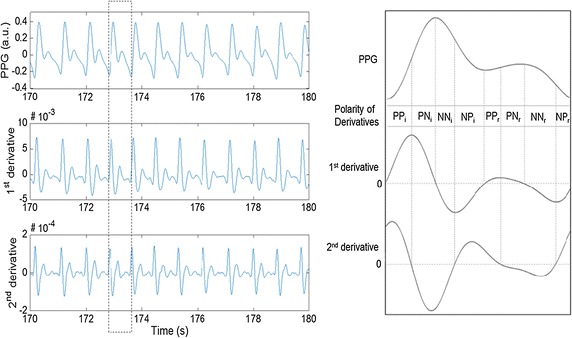
PPG and its derivatives of actual human subjects. Each section was divided from the polarities of derivatives (e.g., PN mean the combination of positive 1st derivative and negative 2nd derivative). First derivative implies the velocity index of blood volume change and second derivative means acceleration of blood volume changes. PP_i_, PN_i_, NN_i_ and NP_i_ are based on incident wave, and PP_r_, PN_r_, NN_r_ and NP_r_ are occurred from reflected wave

Derivative-based analysis provides an evidence for pressure-flow analysis. The velocity of blood volume change, index of flow, could be derived by first derivation [32]. The second derivative of PPG represents the acceleration of blood volume change, and it could be regarded with the rate of pressure gradient change. Figure 2 shows an example of the PPG approximated model waveform about actual human subjects and its derivatives. Beat segmentation was performed prior to derivative analysis, and each beat was extracted between the feet of PPG. Then, it was divided into four different sections by derivatives polarities, first and second character means the polarity of 1st derivative and 2nd derivative, respectively (‘P’ is positive and ‘N’ is negative).

The general PPG waveform is composed with PP–PN–NN–NP combinations. There are two combinations in Fig. 2, and first and second combination is occurred by incident and reflected wave respectively. PP and PN of second combinations could be disappeared in hypertensive subject (Fig. 1b). Though first derivative provides information for the amount of blood volume, it is hard to know the direction of the pressure gradient. Considering that the PPG measures the blood volume changes, first derivative means the increase and decrease of blood volume at a measuring spot. Therefore, the positive and negative value of first derivative means the increase and the decrease of blood volume, respectively. Second derivative provide the rate of blood volume changes which related to existence of opposite pressure. The positive value of second derivative means faster changes of blood volume and negative value means slower changes of blood volume. Fast change means the large pressure difference between inlet and outlet, but slow change means the small pressure difference between both sides. From the pressure-flow relationship, flow is generated by the difference of inlet and outlet pressures. From this analysis, two different combinations of first derivative and second derivative were grouped to identify dominant pressure gradient. Table 1 shows the section information and physical meanings. Dominant pressure means the primary pressure related to blood flow, and sub-dominant pressure means the counter-effective pressure which assists or disturbs blood flow meaningfully.

**Table 1:** Section information and physical meaning for each section

Section	1st derivative	2nd derivative	Blood volume	Rate of bold volume change	Dominant pressure	Sub-dominant pressure	Remarks
PP_i_	Positive	Positive	Increase	Increase (faster)	P_i_	–	Early-systole
PN_i_		Negative		Decrease (slower)	P_i_	P_m_	Late-systole
NN_i_	Negative	Negative	Decrease	Decrease (slower)	P_m_	–	Early-diastole
NP_i_		Positive		Increase (faster)	P_m_	P_o_	Late-diastole
PP_r_	Positive	Positive	Increase	Increase (faster)	P_o_	–	Early-reflection
PN_r_		Negative		Decrease (slower)	P_o_	P_m_	
NN_r_	Negative	Negative	Decrease	Decrease (slower)	P_m_	–	Late-reflection
NP_r_		Positive		Increase (faster)	P_m_	P_o_	

BP is closely related with outward pressure in the vessel, and it is discriminated with propagated pressure in axial direction. P_i_, P_m_ and P_o_ reflect systolic pressure in axial direction, exerted pressure by the walls of blood vessels and diastolic blood pressure (DBP) respectively. Especially, P_m_ is affected by not only the systolic blood pressure (SBP), but also the pulse pressure (PP) which means the difference of the SBP and DBP. P_i_, P_m_ and P_o_ could be described by derivative method. Period A–D′ in Fig. 1 completely corresponds to derivative section PP_i_–NP_r_. From Table 1, NN_i_ section is composed with both the end of systolic effect which related on SBP and the start of the reflected wave effect. This contains the two important points. First, NN_i_ section is composed with both systolic activity (incident wave) and reflected wave, and NN_i_ section is defined by the reflected wave arrival time, which related on angiological parameter such as arterial stiffness and total peripheral resistance, which is closely related to BP change. From the previous study, it is already demonstrated that the reflected wave arrival time is closely related to the vascular characteristics, including pressure-related factors [36–38]. Considering that these characteristics, it is possible to analyze that the NN_i_ section more close to the BP with reflected wave arrival time, especially SBP and PP than any other sections.

Because the length of each segment interval could be affected by subject’s heart rate, the length of a cyclic combination of the segmentation result, *l*PN_i_ + *l*NN_i_ + *l*NP_i_, is used for the normalization. Subject’s height, *h*, is also used as an alternative distance to the approximate path length of the measuring spot. Therefore, we formulated the pressure index (PI) as an arrival time or velocity related parameter of the reflected wave, and it was as follows:

1 $$PI = \left( {\frac{{lNN_{i} + lNP_{i} }}{{lPN_{i} + lNN_{i} + lNP_{i} }}} \right) \cdot h$$

where *h* means height of each subject and *lPP*_i_, *lPN*_i_, *lNN*_i_ and *lNP*_i_ represents the time interval of PP_i_, PN_i_, NN_i_ and NP_i_.

### Experiment

Proposed PI was assessed in 25 young and healthy subjects (9 male and 16 female, mean ages of 22.5 ± 3.1 years, range 17–29 years). No subject had a previous history of cardiovascular disease or was receiving vasoactive drugs. Every experiment was performed in a typical laboratory at an ambient room temperature from 11 a.m. to 6 p.m. Drinking and smoking were prohibited during 24 and 2 h before experiment respectively. Experimental protocol was approved by the Ethic Committee of Wonju Christian Hospital. Subject characteristics are given in Table 2.

**Table 2:** Subject characteristics

Subject no.	Gender	Age	Height (cm)	Weight (kg)	BMI (kg/m^2^)	SBP (mmHg)	DBP (mmHg)	PP (mmHg)	MAP (mmHg)
Subject 1	M	29	178	68.1	21.4	119	72	47	88
Subject 2	M	26	175	63.2	20.6	111	63	48	79
Subject 3	F	26	160.8	57.4	22.2	102	63	39	76
Subject 4	F	21	160.7	59.7	23.1	101	63	38	76
Subject 5	M	29	176.9	70.8	22.6	123	68	55	86
Subject 6	M	24	171.1	67.9	23.1	131	67	64	88
Subject 7	M	20	171.4	65.4	22.2	134	67	67	89
Subject 8	F	22	159.3	47.2	18.5	101	63	38	76
Subject 9	M	25	173.5	77.4	25.7	115	69	46	84
Subject 10	F	22	162.9	54.6	20.5	105	67	38	80
Subject 11	M	23	171.7	71.4	24.2	137	72	65	94
Subject 12	F	25	163.9	65.1	24.2	104	66	38	79
Subject 13	F	19	163.8	63.4	23.6	110	61	49	77
Subject 14	F	21	159.9	60.4	23.6	123	76	47	92
Subject 15	F	19	157.2	52.3	21.1	102	69	33	80
Subject 16	F	22	162.2	65.5	24.7	109	76	33	87
Subject 17	F	23	162.1	59.6	22.6	109	72	37	84
Subject 18	F	19	151.7	53.1	23	108	75	33	86
Subject 19	F	19	153.9	51.4	21.7	96	58	38	71
Subject 20	M	24	169.7	59.5	20.6	131	67	64	88
Subject 21	F	21	159.6	56.1	22	103	60	43	74
Subject 22	F	21	153.2	48.7	20.7	93	65	28	74
Subject 23	F	19	157.1	40.6	16.4	103	65	38	78
Subject 24	F	20	156.6	57	23	113	75	38	88
Subject 25	M	23	162.8	49.5	18.6	108	68	40	81
Mean		22.5	163.8	59.4	22.0	111.6	67.5	44.2	82.2
SD		3.0	7.6	8.7	2.1	12.1	5.0	11.0	6.2
Min		29	178	77.4	25.7	137	76	67	93.7
Max		19	151.7	40.6	16.4	93	58	28	70.7

PPG was measured by MP150 (Biopac™ Inc., USA) on left index finger by TSD100B, plethysmography transducer. Omron HEM-907 was used for BP measurement. PPG measurement system includes a 0.05 Hz single pole roll-off high pass filters, 10 Hz low pass filter and 60 Hz notch filter for noise reduction. Amplifier specification is as follows; output range: ±10 V, noise voltage: 0.5 µV_rms_.

Every data was measured in the supine position. Both PPG and BP were measured at the left hand, and BP was measured before and after PPG measurement. After BP measurement cuff was removed to prevent any occlusion of vessel. Before signal acquisition, every subject had 5-min relaxation period in the supine position to allay subject’s excitations. PPG was measured with 5-min length, and BP was measured before and after PPG measurement and averaged. MATLAB 2008b (The MathWorks, Inc., Natick, MA, USA) and SPSS (ver. 12.0, SPSS Inc., IL, USA) was used for signal analysis and statistical analysis respectively.

## Results

### Sectionization by derivatives

Before sectionization, preprocessing, feature detection and pulse shape extraction were carried out. In preprocessing stage, PPG waveform was filtered using 2nd order Butterworth bandpass filter which passband is 0.5–10 Hz to remove high-frequency noise and low-frequency noise like motion artifact or respiratory movement noise. Then, we used adaptive threshold peak detection method for feature detection [39]. Because those sections are defined within pulse duration and that pulse duration is based on a maximum diastolic point, we detected lower peaks of PPG waveform before dividing sections.

Ten beat segments were randomly selected from each subject for sectionization, and section lengths were ensemble averaged to calculate average section length. The *Pearson*’s correlation was used to find a correlation between at least two continuous variables, and it was calculated between PI and SBP, DBP, PP and MAP for PI evaluation. PP and MAP are described in (2) and (3) respectively.

2 $$PP = SBP - DBP$$

3 $$M{\text{AP}} = {\text{DBP}} + \frac{ 1}{ 3}{\text{PP}}$$

**Fig. 3:**
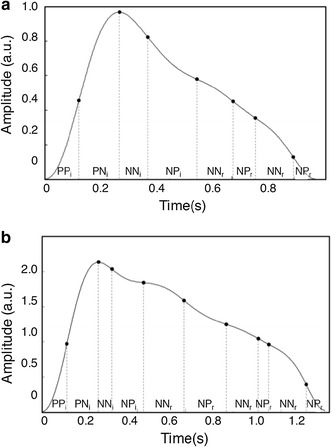
Sectionization results of PPG waveform for actual human subjects. **a** PPG from hypotensive subject (SBP/DBP: 96/58 mmHg, subject no. 19), **b** PPG from hypertensive subject (SBP/DBP: 137/72 mmHg, subject no. 11)

In sectionization, different numbers of sections were found in different subjects. These differences are also found in each beat of the same subject; however variation has been just a little and the number of sections from the incident wave was fixed at four. Figure 3 shows sectionization results of actual human subjects for the (a) hypotensive (systolic pressure/diastolic pressure: 97/60 mmHg, subject no. 21) and (b) pre-hypertensive (systolic pressure/diastolic pressure: 137/72 mmHg, subject no. 16). It was ignored that reflected wave section because the approximated model which is consisted with a single incident and reflected wave was adapted.

### Statistical analysis

**Fig. 4:**
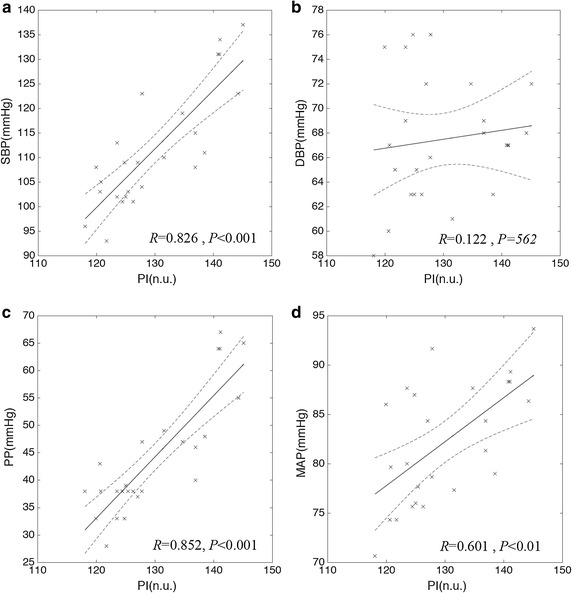
Graph to show correlation between blood pressures and derived pressure index (PI). **a** Systolic blood pressure (SBP) versus PI, **b** diastolic blood pressure (DBP) versus PI, **c** pulse pressure (PP) versus PI, **d** mean arterial pressure (MAP) versus PI; *times symbol* is data point; *solid line* is linear regression curve; *dashed line* is 95% confidence interval (*n.u.* null unit)

Both sectional modeling and PI correlation coefficient were analyzed to verify our hypothesis related to pressure components. It was not found the statistically significant correlation in sectional analysis except in NN_i_ section. In this paper, hypothesis means estimate of PI by modeling. Thus, separate control group verification is not necessary. In NN_i_ section, SBP and PP, and MAP satisfied *P* < 0.01 and *P* < 0.05 respectively. Meaningful correlation coefficient in NN_i_ section was represented as −0.501 (SBP) and −0.548 (PP). Calculated PI shows the highest correlation coefficient against each sectional length. A correlation coefficient of PI shows 0.826 (SBP), 0.852 (PP) and 0.601 (MAP), and all of these have meaningful statistically significance (*P* < 0.01). Figure 4 represents the correlation between PI and pressures [(a) SBP vs. PI, (b) DBP vs. PI, (c) PP vs. PI, (d) MAP vs. PI]. Solid line means linear regression of plotting data and 95% of confidence intervals. We used the ordinary least squares-based linear regression which minimizes the sum of squared residuals, and confidence interval was calculated with (4) where $\bar{y}$ is an average estimated value, *n* is a number of samples and *σ* is a standard deviation of the estimated value.

4 $$95\% \,CI = \bar{y} \pm 1.96\frac{\sigma }{\sqrt n }$$

## Discussion

### Hypothesizes validation

From the analytical results, it was appeared that (1) sectional ratio of NN_i_ is statistically significantly related on BPs such as SBP and PP, but the relationship was not found in other section, (2) proposed PI is statistically significantly correlated with BPs, and it reflects SBP and PP more than other pressures. These results correspond with our hypothesizes respectively: NN_i_ section dominantly reflects SBP and PP, and the combination of incident and reflected wave morphology is affected by BP. NN_i_ length tended to show shorter sectional length by increasing of SBP and PP. From Fig. 1 and Table 1, NN_i_ is defined by ‘decrease blood volume’ and ‘decrease blood volume change velocity’. These mean that the period of NN_i_ is started at the moment that outflow becomes more than inflow and is finished the moment that the outflow velocity is decreased by opposite pressure wave. Here, the moment that the amount of outflow excess inflow could be happened at the maximum systolic pressure and an opposite pressure wave which decreases the outflow velocity could be regarded as a reflected wave. Therefore, it could be postulated that NN_i_ indirectly reflects the time interval between maximum systolic pressure and that the reflected wave becomes effective.

Considering that NN_i_ is determined from the moment that outflow becomes more than inflow to the moment that the outflow velocity is decreased by opposite pressure wave, it could be postulated that NN_i_ indirectly reflects the time interval between maximum systolic pressure and that the reflected wave becomes effective. From the previous study, it is already demonstrated that reflected wave arrival time is closely related to vascular characteristics, including pressure-related factors [36–38], and this research shows the proposed PI based on sectioned waveform could be a statistically significant marker of BP in normotensive subject. Considering the definition of NN_i_ length, the faster reflected pulse wave velocity becomes, the shorter NN_i_ length is presented. In other words, NN_i_ becomes shorter when reflected wave is arriving earlier, and NN_i_ and reflected wave velocity has a reciprocal relationship. Therefore, it is a reasonable result that NN_i_ which implies a reflected wave velocity shows negative correlation coefficient, which means the higher BP, the shorter section interval. However, in Table 3, low correlation coefficient (*R* = −0.501) between NN_i_ and SBP represents the uncertainty of PPG morphology. PPG morphology, especially sectional length could be affected by heart rate and measuring distance, and this means the normalized parameter should be needed. Therefore, the PI was normalized by a cyclic combination of segmented waveform. PI is formulated by multiplying NN_i_ ratio in the incident wave and height to describe the wave dispersion by measuring distance. In this procedure, we define NN_i_ as a specified region of reflected waveform and PI as a kind of index term of reflected wave arrival time. We also adapt h as an approximated path length of subject, and reflect to define PI. Consequently, PI shows improved correlation with SBP and PP, and it provides the appropriateness of our hypothesizes. There is an additional considerable point is that the high correlation coefficient of PP. In the result of correlation test, PP and SBP show significance with PI, and correlation coefficient of PP is slightly higher than SBP. This result might be interpreted as that PI reflects well PP and that high correlation of SBP comes from SBP = PP + DBP. However, this postulation should be investigated with arterial stiffness and needs to be validated by further researches.

**Table 3:** Correlation coefficient between each segment and pressures

	SBP	DBP	PP	MAP
PP_i_	−0.092	−0.258	0.017	−0.199
PN_i_	−0.330	−0.412*	−0.174	−0.436*
NN_i_	0.501**	−0.005	0.548**	0.327
NP_i_	0.206	−0.181	0.309	0.036
h	0.703**	0.171	0.736**	0.514**
PI	0.826***	0.122	0.852***	0.601**

*** *P* < 0.001; ** *P* < 0.01; * *P* < 0.05

In DBP case, suggested method could not show statistically significance results. BP estimation of this paper is based on an approximated model which consists of single incident and reflected wave. Here, incident and reflect wave is naturally generated by the pulsatile activity of heart and peripheral reflection. In other words, there is a little ambiguity in DBP estimation using pulsatile components because pulsatile is more closely related to the systolic activity than diastolic activity. Therefore, in this literature, the correlation value of DBP was not high compared with the correlation value of SBP.

### Limitations

The purpose of this research is for the intermittent use of BP estimation rather than continuous BP monitoring. Therefore, we randomly sampled individual pulses of signal which recorded in resting condition, then compared estimated pressure-related values with average BP of pre- and post-recording. It means that the proposed method is focused on the tendency of BP but it is not validated in analyzing the respiratory variability which is observed in continuous BP monitoring.

This study has other important limitations, however, mostly stemming from its small subject size. PPG morphology could be affected by not only BP, but also aging [40], vessel stiffness [41, 42], cardiovascular diseases and other hemodynamic properties. Moreover, it could be varied by vasoactive drugs or endothelial function [3, 43]. Especially, factor which closely related to the arterial stiffness needs to be investigated sophisticatedly because it could effect on the wave reflection; therefore, much larger sample set including a wide range of age and BP would be needed in the future research. In Allen’s review, we can find the various factors in changing PPG morphology [30]. In this literature, we only focused on the macroscopic morphology of PPG waveform based on reflected wave analysis. Reflected wave naturally includes the angiological characteristics like vessel stiffness, and this approach may have a meaning as a simple approach to BP. However, this approach not yet provide a sophisticated estimation for the separated analysis of various subject’s physiological characteristics. For example, subjects who have the cardiovascular diseases and receive vasoactive drugs were excluded in this paper. Therefore, proposed method may not be adaptable to cardiac and vascular patients, and it should be solved by detailed and specified parameter centered experiment such as patient group test pharmacological test.

Also, the morphology of PPG waveform could vary due to other physiological factors, such as spring clip pressure, cardiac output, airway pressure, venous pressures and fluid responsiveness. Changes of photoplethysmographic morphology could be interpreted in terms of earlier arrival of a pressure wave reflected from the peripheral circulation [7, 12]. Therefore, it should be studied about the interaction between reflected wave and variation factors for practical application using the proposed index in the future works.

### Concluding remarks

Results from the present study highlight the PPG morphological analysis based on pressure-flow relationship and correlation analysis between BP and derived parameter. It is appeared that proposed estimation index is statistically significantly correlated (*P* < 0.05) with SBP (*R* = 0.826), PP (*R* = 0.852) and MAP (*R* = 0.601). This is a novel study to analyze between PPG morphology and BP without any other assistive devices, and it may be applied to further researches based on PPG and BP analysis. Unfortunately, current study could not explain clearly to the DBP and which pressure component would most suitably be estimated using the technique. Moreover, this study has some insufficiency to use in practice, which is stemmed from a limited subject group. However, we expect that this study could be an effective way of BP estimation by additional angiological and pharmacological experiment. Currently, cuff less BP measurement technique, PTT-based measurement technique, has been well studied however; it requires multi-devices which are ECG and PPG. Moreover, PTT-based BP estimation has a limitation for SBP and DBP estimation because it could provide only a variable, PTT. Thus, if an additional parameter like our proposed parameter adapted to an existing method PTT, BP estimation would be enhanced. Also, our further study would make a possible to estimate BP with only PPG. We strongly believe that our proposed study could provide potential techniques for the more accuracy BP estimation and the more efficiency for BP measurement.

## Abbreviations

ABP: arterial blood pressure
BP: blood pressure
ECG: electrocardiogram
DBP: diastolic blood pressure
MAP: mean arterial pressure
MEMS: micro-electro mechanical systems
PAT: pulse arrival time
PI: pressure index
PPG: photoplethysmography
PTT: pulse transmit time
SBP: systolic blood pressure
SDPTG: second derivative of the photoplethysmogram waveform
